# Visualizing the invisible: novel approaches to visualizing bacterial proteins and host-pathogen interactions

**DOI:** 10.3389/fbioe.2024.1334503

**Published:** 2024-02-13

**Authors:** Moirangthem Kiran Singh, Linda J. Kenney

**Affiliations:** ^1^ Department of Biochemistry and Molecular Biology, University of Texas Medical Branch, Galveston, TX, United States; ^2^ Sealy Center for Structural Biology, University of Texas Medical Branch, Galveston, TX, United States

**Keywords:** host-pathogen interactions, protein labeling, bacterial proteins, imaging techniques, super-resolution microscopy, genetic code expansion

## Abstract

Host-pathogen interactions play a critical role in infectious diseases, and understanding the underlying mechanisms is vital for developing effective therapeutic strategies. The visualization and characterization of bacterial proteins within host cells is key to unraveling the dynamics of these interactions. Various protein labeling strategies have emerged as powerful tools for studying host-pathogen interactions, enabling the tracking, localization, and functional analysis of bacterial proteins in real-time. However, the labeling and localization of *Salmonella* secreted type III secretion system (T3SS) effectors in host cells poses technical challenges. Conventional methods disrupt effector stoichiometry and often result in non-specific staining. Bulky fluorescent protein fusions interfere with effector secretion, while other tagging systems such as 4Cys-FLaSH/Split-GFP suffer from low labeling specificity and a poor signal-to-noise ratio. Recent advances in state-of-the-art techniques have augmented the existing toolkit for monitoring the translocation and dynamics of bacterial effectors. This comprehensive review delves into the bacterial protein labeling strategies and their application in imaging host-pathogen interactions. Lastly, we explore the obstacles faced and potential pathways forward in the realm of protein labeling strategies for visualizing interactions between hosts and pathogens.

## 1 Introduction

Infectious diseases pose significant risk to human wellbeing, resulting in widespread morbidity and mortality worldwide. Pui et al. (2011) The emergence of infectious diseases can be attributed to intricate interactions between invading microorganisms and the host immune system. Mølbak (2005) To effectively combat infectious diseases, it is crucial to grasp the intricate molecular mechanisms at play during these interactions. Gram-negative pathogens such as *Salmonella, Pseudomonas aeruginosa, Shigella,* EPEC*,* and *Yersinia,* among others, transmit their virulence factors or effectors into host cells via needle-like complex nanomachines such as type-III secretion systems (T3SS) or Type-IV secretion systems, releasing dozens of effectors into the cell in a highly regulated manner Erhardt et al. (2010), Costa et al. (2015), LaRock et al. (2015), Jennings et al. (2017), Galán and Waksman (2018), Kenney (2019), Liew et al. (2019). This strategic maneuver enables bacteria to manipulate host cellular processes, creating a favorable environment for survival. For instance, *Salmonella* utilizes two T3SS that are encoded in two distinct genomic regions known as *Salmonella* pathogenicity island 1 (SPI-1) and *Salmonella* pathogenicity island 2 (SPI-2), which facilitate the delivery of −50 effectors into host cells. These mixture of effectors initiate substantial alterations in endosomes, leading to the creation of highly dynamic and extensive tubular membrane structures known as *Salmonella*-induced filaments (SIFs). While the SPI-1 region encodes the T3SS and effectors that facilitate invasion, the SPI-2 encoded T3SS and effectors promote survival and replication within the *Salmonella*-Containing Vacuole (SCV) Erhardt et al. (2010), Costa et al. (2015), LaRock et al. (2015), Jennings et al. (2017), Galán and Waksman (2018), Kenney (2019). Understanding the specific functions of these effector proteins enables a deeper understanding of the virulence of *Salmonella* and other Enterobacteriaceae. Identifying and characterizing effector proteins and understanding their functions through traditional biochemical analysis, sequence comparisons, and structural studies have been invaluable. However, these methods do not unveil details about how, when, or why an effector protein influences the infection process. Imaging the secretion process itself and tracing the pathways of translocated effectors is a promising strategy for revealing the operation of these effectors. However, it is important to note that labeling and localizing secreted T3SS effectors within host cells has proven to be technically challenging, as discussed by us in another review. Singh and Kenney (2021).

In the past, effector proteins were individually over-expressed in host cells and subsequently localized using immunofluorescence techniques Ohlson et al. (2008), McGourty et al. (2012). This approach has its drawbacks in disrupting effector stoichiometries and altering their copy number. Furthermore, the incorporation of epitope markers can be challenging and the use of antibodies often results in background artifacts Schnell et al. (2012). The conventional and widely adopted method for tracking and localizing a protein of interest within cells involves expressing it fused with a fluorescent protein (FP), such as GFP, mCherry or its variants. However, there is a caveat when it comes to T3SS secreted effector proteins. Attaching a bulky fluorescent protein such as GFP directly to an effector not only hinders its secretion, but also interferes with the functioning of the T3SS machinery Akeda and Galán (2005). Transfection enables researchers to introduce effector-GFP combinations into host cells, but localization of host-expressed effectors can differ from effectors translocated via the T3SS. For example, PipB2-GFP expression concentrated around the cell periphery and near the nucleus, while the T3SS-translocated PipB2-GFP (utilizing a split-GFP system) was predominantly found within the tubular network (SIFs) Van Engelenburg and Palmer (2010). This discrepancy emphasizes the importance of expressing tagged effectors in bacterial cells, as opposed to relying on transfection in host cells.

**FIGURE 1:**
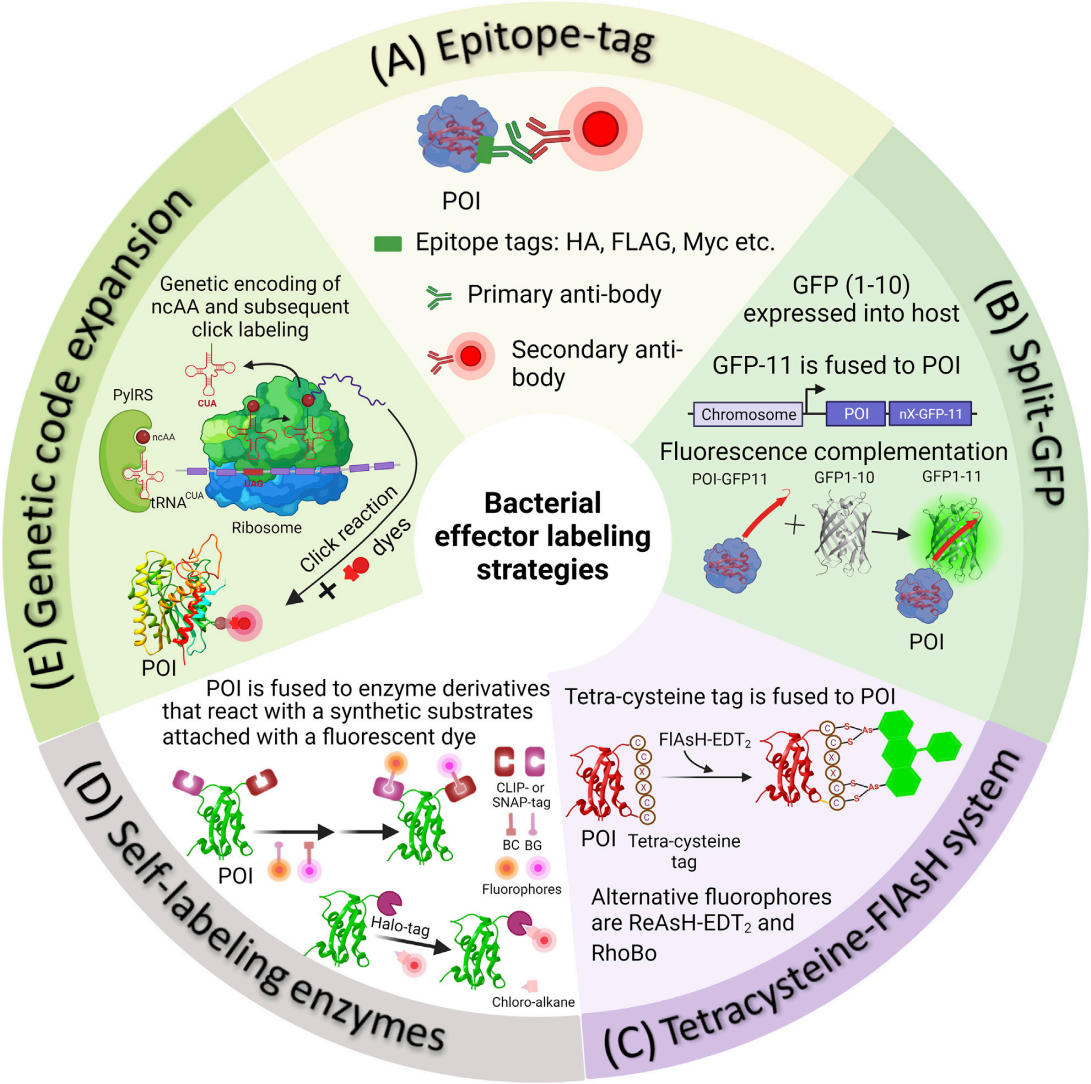
Methods of fluorescently tagging effector proteins of interest. **(A)** Epitope tags can be attached genetically to either the N- or C-terminus of a protein of interest. Following this, *Salmonella* infected cells are fixed and subjected to immunostaining using appropriate primary and secondary antibodies. **(B)** In the split-GFP method, the β-barrel, referred to as GFP-11, is genetically fused with a POI. Simultaneously, the complementary fragment comprising the initial ten strands of GFP (GFP1-10) is introduced into the host cell externally. Detecting translocated effectors in the host cell occurs when the two fragments spontaneously reassemble, resulting in the formation of a complete GFP molecule. **(C)** In the 4Cys‐FlAsH system, FlAsH bioconjugation takes place when FlAsH-EDT2 reacts with the tetracysteine motif genetically fused into the effector POI. **(D)** In self-labeling enzyme methods, genetically encoded markers like HaloTag, SNAP-tag, or CLIP-tag are employed to label the proteins of interest, which are then expressed in *Salmonella*. Detection of secreted effectors in host cells is made possible by using a fluorophore substrate that contains both the fluorophore and a chemical component promoting covalent binding with the marker. **(E)** The diagram illustrates the process of SPI-2 effector labeling using GCE. In the bacterial cell, the plasmids containing the gene of the POI (purple mRNA), an orthogonal suppressor tRNA (in red), and an aminoacyl synthetase (in green) are introduced into the bacterial cell. At an appropriate location within the effector gene sequence, a native codon is substituted with an amber stop codon (red in purple mRNA). The ncAA, represented as a dark red circle, is added to the growth medium. The cell absorbs the ncAA, which then enters the cytosol, where it binds to the orthogonal tRNA through the aminoacyl-tRNA synthetase (aaRS). The ncAA-acylated tRNA, equipped with a CUA anticodon, enters the ribosomal machinery and pairs with the corresponding amber codon on the effector mRNA (in purple). This leads to the integration of the ncAA into the effector. The newly synthesized effector is subsequently transported into the host cell through the Type III Secretion System (T3SS). When required, the visualization of secreted effectors occurs through a click reaction between an externally introduced fluorophore and a biorthogonal handle that is part of the non-canonical amino acid integrated into the effector POI.

Alternatives to GFP are constantly being developed to elucidate the mechanisms underlying effector protein translocation and localization within the host Young and Palmer (2017), Braet et al. (2022). Therefore, within the context of *Salmonella*-related studies, this review aims to emphasize methodologies that have been devised for the identification and surveillance of effector protein translocation, verification of their involvement in virulence, and visualization of their localization within host cells (See Figure 1). We begin by describing how to create active fluorescent protein fusions encoded on the chromosome, which enables the use of photoactivated localization microscopy (PALM) study and analysis. Next, we discuss commonly used strategies for precise labeling of proteins of interest (POIs) and subsequent imaging of bacterial secreted effectors in host cells. More specifically, we will focus on techniques based on epitope tags, fluorescence complementation via split‐GFP, direct labeling of effectors using the tetracysteine‐FlAsH/ReAsH system, and self-labeling enzymes. We provide a thorough evaluation of the advantages and limitations of each approach. Finally, we delve into greater detail of the latest advancements in genetic code expansion techniques for labeling purposes, as well as the application of super-resolution imaging to visualize bacterial secreted effectors within host cells. Table 1 provides an overview of protein labeling methods used to monitor effector protein translocation.

**TABLE 1:** Overview of protein labeling methods to monitor effector proteins.

Protein labeling methods	Size of tags (kDa)	Translocation	Real-time detection of secreted effectors	Representative effectors studied [references]	Advantages and disadvantages of the labeling methods	
					Advantages	Disadvantages
Fluorescent protein fusion	∼27	X	X	SifA, SifB Ohlson et al. (2008), McGourty et al. (2012)	(1) highly specific; (2) easy to express as protein fusions with target effectors; (3) no need to add dyes from outside cells; (4) wide range of colors and properties	(1) large size of protein fusion (PF) blocks T3SS and hinders effector secretion. Therefore, PF needs to be individually expressed within host cells; (3) localization of host-expressed effectors differs from effectors translocated via the T3SS.
				PipB2 Van Engelenburg and Palmer (2010) individually over-expressed into host cells		
Epitope-tags	<1-2	✓	X	*Salmonella* Typhimurium	(1) small size and minimal interference with protein function; (2) wide range of antibodies accessible with a diverse array of dyes	(1) requires permeabilization and fixation for intracellular targets; (2) potential cross-reactivity and background fluorescence
				SPI-1 and SPI-2 effectors Cain et al. (2004), Brawn et al. (2007), Gao et al. (2018)		
Split-GFP	27	✓	✓	*Salmonella* Typhimurium	(1) small size of GFP fragments; (2) no exogenous ligand required; (3) long term visualization in real-time; (4) signal enhancement by using tandem tags	(1) slow and irreversible reconstitution; (2) complementation kinetics limit use to 15–30 min post infection or after. (3) moderate sensitivity due to limited signal intensity; (4) potential interference of effector function due to tag
				PipB2, SteA Van Engelenburg and Palmer (2010), Young et al. (2017)		
				*Pseudomonas* syringae AvrB, AvrRps4 Park et al. (2017)		
4Cys-FlAsH	<1	✓	✓	*Salmonella* Typhimurium SopE2, SptP Van Engelenburg and Palmer (2008)	(1) small label size; (2) high affinity and specificity of the dye-tag; (3) real-time kinetics; (4) signal enhancement by using tandem tags	(1) cytotoxic; (2) limited to the very early translocated effectors for visualizing translocation; (3) low sensitivity due to limited signal intensity and background fluorescence; (4) potential interference of the effector function due to tag
				*S. flexneri* IpaB/C Enninga et al. (2005)		
Self-labeling enzymes	20-33	✓	✓	*Salmonella* SPI-1 and SPI-2 effectors Göser et al. (2019)	(1) fast formation of stable covalent bonds; (2) various fluorophores can be coupled; (3) non-toxic and cell permeable; (4) pre- and post-labeling possible	(1) large size of enzyme tags may perturb localization and function of effector protein; (2) reduced translocation of fusion proteins (3) non-specific labeling is possible. SNAP-tag substrate shows high background fluorescence in host cells
				*Yersinia* pestis YopM Göser et al. (2019)		
Genetic code expansion (using chemically reactive ncAAs)	<1	✓	X	*Salmonella* Typhimurium	(1) site-specific and covalent labeling; (2) compatible with various synthetic fluorophores; (3) small and non-perturbative	(1) requires exogenous addition of ncAAs and orthogonal tRNA-synthetase pair; (2) click reaction is required and most dyes are cell impermeable; (3) non-specific labeling is possible
				SifA, SseJ, SsaP Singh et al. (2021), Singh and Kenney (2023)		
Genetic code expansion (using intrinsically fluorescent ncAAs)	<1	✓	--	*Salmonella* Typhimurium	(1) highly specific; (2) small and non-perturbative; (3) no need to add fluorescent dyes externally	(1) too dim and too blue for fluorescent imaging; (2) requires two-photon fluorescence microscopy for most of existing fluorescent ncAAs
				SseJ Singh et al. (2021), Singh and Kenney (2023)		

## 2 Construction of an active, chromosomally expressed photoactivatable fusion protein for single-molecule localization microscopy

Fluorescence microscopy is an established technique for observing and studying biological structures and processes within living organisms. It is non-invasive and enables real-time monitoring of a wide variety of biological processes. Nevertheless, the capabilities of fluorescence microscopy are limited by the laws of wave optics, which dictate that the resolution is roughly limited to half the wavelength of visible light (λ⁄(2NA), where λ: detecting wavelength; NA: numerical aperture) Abbe (1873). This poses a significant challenge, as numerous subcellular structures have dimensions smaller than 100 nm. Conventional fluorescence microscopy involves the simultaneous excitation of a large number of fluorophores, which causes the point spread functions (PSFs) of these fluorophores to overlap, resulting in a fuzzy image. Numerous biological structures have dimensions that are smaller than the PSFs, rendering them outside the reach of traditional fluorescence microscopy. While electron microscopy has partially addressed this issue, it is not without its own constraints pertaining to sample preparation and contrast. In recent years, there have been significant advancements in fluorescence microscopy techniques, resulting in a transformative impact on the field. These unique approaches have enabled the achievement of near-molecular resolution, marking a significant milestone. Many of these techniques, collectively known as “single-molecule localization microscopy” (SMLM), rely on the iterative imaging of limited and random subsets of fluorophores inside a specimen (Betzig et al., 2006; Lelek et al., 2021). The localization of active fluorophores is achieved by determining the centers of their point spread functions, which are then used to generate a super-resolved image. Experimentally, all fluorophores are first set to their inactive (off) state. Following this step, fluorophores are selectively activated at low densities by precisely modulating the intensity of the photoactivating light, ensuring that only a limited number of fluorophores become visible in each frame of the wide-field microscope. A range of SMLM-based methodologies has emerged: stochastic optical reconstruction microscopy (STORM), direct STORM (dSTORM), photoactivated localization microscopy (PALM), fluorescence photoactivation localization microscopy (FPALM), ground-state depletion microscopy (GSDIM) and more (Rust et al., 2006; Folling et al., 2008; Heilemann et al., 2008; Lemmer et al., 2008; van de Linde et al., 2011; Turkowyd et al., 2016; Vojnovic et al., 2019). Central to this approach is the use of fluorophores that can switch between their “on” and “off” states in a controlled manner, allowing straightforward and accurate localization. The precision of molecular localization is approximately inversely related to the square root of the acquired photon count. Therefore, to acquire high resolution images, a large photon yield in the active state is necessary. Although numerous such fluorophores are available, they each come with their own set of limitations. Consequently, a critical decision in the planning of an SMLM experiment revolves around selecting the most suitable fluorophore for the task.

One straightforward approach to ascertain the spatial distribution of a protein of interest via SMLM involves genetically fusing the protein to a suitable fluorescent protein. Historically, PALM techniques employed photoactivatable fluorescent proteins (PAFPs) or photoswitchable fluorescent proteins (PSFPs). PAFPs such as photoactivatable GFP (PAGFP), photoactivatable monomeric Cherry (PAmCherry), and PATagRFP, are a type of fluorescent protein that exhibit fluorescence that can be modified by a light-induced chemical reaction. They can be activated from a non-fluorescent state to a fluorescent state. This makes PAFPs useful for selective optical markers, allowing for the tracking of protein trafficking, diffusion, and turnover. In contrast, PSFPs represent a distinct group of proteins capable of switching between two distinct fluorescent colors. One such PSFP, PSmOrange, is orange but shifts to a far-red hue following irradiation with blue-green light. The far-red color that PSmOrange attains after photoswitching is especially advantageous for deep-tissue imaging. This advantage arises from the deeper tissue penetration capability of far-red light, facilitating enhanced visualization of biological structures. Readers are encouraged to consult comprehensive reviews, which provide an in-depth examination of the pros and cons of currently available PAFPs and PSFPs, and super-resolution imaging using PAFPs (Lippincott-Schwartz and Patterson, 2009; Finan et al., 2013; Durisic et al., 2014; Nienhaus and Ulrich Nienhaus, 2014; Wang et al., 2014). When utilizing genetically encoded fluorescent protein fusions, several critical parameters should be considered. In this section, we will delve into how specific challenges have been effectively addressed, using examples to enhance the utility of fluorescent protein fusions for imaging applications.

The use of fluorescent proteins has various advantages, owing principally to their ability to form genetic fusions with specific targets. Nonspecific background labeling is absent when using the fluorescent protein fusion method, in contrast to affinity labeling techniques employing antibodies or small molecules. This can be significant when studying individual molecules, as their fluorescence may be susceptible to interference from background fluorescence. Although organic dyes have superior photophysical characteristics, effective labeling of intracellular proteins using organic dyes can be problematic due to their poor permeability compared to protein fusions. Thus, organic fluorescent dyes are more suitable for use with fixed cells (Kipper et al., 2017). Nevertheless, PAFPs also have numerous limitations. PAFPs typically emit a lower number of photons per “burst” in comparison to fluorescent dyes, which often results in the generation of images with low resolution. The fusion can also destabilize, mis-localize or functionally impair the target protein or drive oligomerization. Despite extensive efforts in creating soluble monomeric variants, the issue of oligomerization remains a significant concern since numerous PAFPs are produced from parent proteins that normally exist as dimers or tetramers. It is strongly advised to employ monomeric forms of PAFPs to prevent undesirable aggregation of the target protein.

It is imperative to emphasize that the localization of a protein does not serve as a predictive indicator of its function. Fluorescent protein fusions can affect target protein function and localization by disrupting function. For instance, the interaction between the bacterial cytoplasmic protein CheY and the switch proteins associated with the flagellar motor plays a crucial role in regulating rotational direction during bacterial chemotaxis. Introduction of a CheY-tdEos fusion construct in a strain lacking *cheY* did not successfully restore chemotaxis (Greenfield et al., 2009). Previous studies demonstrated that C-terminal fusions of the transcription factor OmpR altered its ability to bind to DNA (Slauch et al., 1988; Perkins et al., 2013; Quinn et al., 2014; Shimada et al., 2015). In *Salmonella*, the SsrA/B two-component system activates SPI-2 encoded effectors in response to acid pH. N-terminal fusions of *ssrB* and C-terminal fusions of *ssrA* to PAmCherry, when encoded on the chromosome, were functionally active and capable of activating SPI-2 gene transcription. In contrast, C-terminal fusions of *ssrB* and N-terminal fusions of *ssrA* were inactive (Liew et al., 2019). The protein SsaP plays a crucial role in facilitating the transition from the secretion of substrates involved in injectisome assembly to the secretion of SPI-2 effectors, hence enabling a switch in secretion specificity. Attaching PAmCherry to the C-terminus to create a fusion protein led to protein cleavage, consequently impeding the visualization of SsaP (Singh et al., 2021). Hence, care must be exercised when creating fusion proteins and assessment of their functionality is imperative to determine their ability to substitute the original protein within the cellular context.

Previous research has shown cases in which fusions are not well-tolerated at either terminus but remain stable inside an interior flexible region of the protein under investigation (Giraldez et al., 2005). The choice between N- or C-termini and the decision to include a fluorescent protein within a flexible loop should be determined by functional demands. Indeed, when both N-terminal and C-terminal fusions are not fully functional and are confounded with imaging artifacts, the solution may lie in the implementation of a sandwich fusion. Studies of the bacterial actin homologue MreB provide a cautionary tale (Taghbalout and Rothfield, 2007; Jennings et al., 2011). When yellow fluorescent protein (YFP) was fused to its N-terminus, MreB formed filaments and extended helices in *E. coli*. Yet, no evidence of MreB helices was observed in unlabeled cells from tomograms using cryo-electron microscopy (Swulius and Jensen, 2012). Addition of YFP to the N-terminus disrupted function, whereas the MreB-YFP only partially restored the cellular morphology of *mreB* null cells (Shih et al., 2003). Furthermore, extended helices still formed when the YFP marker was replaced with a reversibly photoswitchable enhanced GFP (rsEGFP) fused to the N-terminus (Grotjohann et al., 2011). When mCherry was fused to an internal loop, the resulting MreB-mCherry was phenotypically similar to wild-type, and no filaments were observed (Swulius and Jensen, 2012). These data collectively demonstrate the potential of FP tagging to disrupt biomolecular interactions, sometimes leading to misleading outcomes. The positioning of the protein tag significantly influenced both the function and localization pattern of MreB, underscoring the importance of implementing control experiments to minimize the possibility of labeling artifacts. The findings from subsequent super-resolution imaging studies have demonstrated that MreB has a distinct organizational pattern within the cell. Instead of forming vast filaments, MreB is observed to be organized into shorter filaments and patches that undergo circumferential movement (Domínguez-Escobar et al., 2011; Garner et al., 2011; Van Teeffelen et al., 2011).

The fusion of a fluorescent protein could potentially impede the functionality of the protein of interest by causing steric hindrance and interfering with proper protein folding. The application of a linker can sometimes overcome these problems (English et al., 2011; Chen et al., 2013; Foo et al., 2015; Liew et al., 2019). OmpR is best known for its role in regulating the transcription of outer membrane porin genes *ompC* and *ompF* in response to osmotic and acid stress (Kenney and Anand, 2019). Structurally, it consists of two domains: an N-terminal phosphorylation domain (structurally homologous to CheY) and a C-terminal DNA binding domain. To investigate the spatial distribution and localization of OmpR within cells, the C-terminus of OmpR was directly joined to GFP (Batchelor and Goulian, 2006). Activation of the target gene *ompC* was only 20% compared to wild-type OmpR. A comparable degree of functionality was also observed with a PAmCherry fusion (Foo et al., 2015). Incorporation of a flexible linker between the OmpR C-terminus and PAmCherry resulted in a functional hybrid protein with activation >70%. The OmpR-PAmCherry fusion protein was sufficiently active with a linker of 16 amino acids, while an OmpR-mEos fusion protein required a 40 amino acid linker (Foo et al., 2015). Lastly, it is advisable to perform super-resolution studies at levels of physiological expression whenever feasible. For localization and visualization of bacterial effectors secreted through the T3SS, bulky fusion proteins such as GFP must be avoided, as they jam the T3SS (Singh et al., 2021; Singh and Kenney, 2021). In the next section, we will provide more details of visualizing bacterial secreted effectors.

## 3 Protein labeling strategies to visualized secreted effectors

Monitoring bacterial secreted effectors is technically challenging. These challenges arise from the translocation of secreted effectors through the T3SS. Chaperones in the bacterial cytosol facilitate the partial unfolding of effectors and their transportation to the T3SS (Akeda and Galán, 2005; Tsai et al., 2015). Notably, FPs or PAFPs cannot be used for this purpose due to their high thermodynamic stability (Radics et al., 2014; Singh et al., 2021; Singh and Kenney, 2021). Consequently, alternative labeling methods are required to tag and visualize T3SS-translocated bacterial effector proteins. In this section, we will discuss some of the widely used methodologies with a focus on *Salmonella* secreted effectors (Figure 1; Table 1).

### 3.1 Detection of bacterial secreted effectors via epitope tags

In the past, the analysis of bacterial secreted effectors involved using monoclonal antibodies against these effectors in either bacterial culture supernatants or infected cell lysates (Collazo and Galán, 1997). Due to the limited availability of effective antibodies, researchers often had to resort to utilizing endogenous epitope tags to perform immunofluorescence labeling of the protein of interest. Epitope tagging directly fuses the epitope to the target effector protein. These short epitope tags, typically consisting of 8–18 amino acid residues, such as FLAG (sequence—DYKDDDDK), HA (sequence - YPYDVPDYA), myc (sequence—EQKLISEEDL), M45 (sequence—MDRSRDRLPPFETETRIL) etc., offer the advantage of minimizing potential disruptions to the structure and biological function of the fused target protein (Waugh, 2005; Young et al., 2012). These tags can then be fluorescently labeled using high-affinity monoclonal antibodies specific to the epitope, many of which are commercially available (see Figure 1A for the labeling scheme).

Immunofluorescence has been vital to understanding the diversity of *Salmonella*-containing vacuole (SCV)-associated filaments and the roles of specific effector proteins in *Salmonella* infection (Schroeder et al., 2011). Although it was feasible to produce antibodies against the immunogenic SipA and SipC, effector proteins such as SopB, SptP, SopE, and SopE2 were instead detected using C-terminal fused FLAG tags, revealing their localization at bacterial attachment sites leading to membrane ruffling and actin reorganization, at the cell periphery, along SCV membrane and tubular networks (Cain et al., 2004; Stévenin et al., 2019). Immunofluorescence provided valuable insights into the functional implications of SopB in the recruitment of sorting nexin-1 to the *Salmonella*-containing vacuole (SCV) (Bujny et al., 2008), involvement of SptP in facilitating inter-organ dissemination of *Salmonella* in mouse models (Choi et al., 2013), and the participation of SifA, SseJ, SseG, and SseF in the process of tubule formation (Beuzón et al., 2000; Kuhle et al., 2004; Birmingham et al., 2005; Gao et al., 2018; Singh et al., 2021). The functions of SifA, SseJ, and SopD2 in preserving the structural integrity of SCV membrane have been elucidated (Brumell et al., 2002a; Ohlson et al., 2008). The use of epitope-tagged versions of SipA and PipB2 revealed collaborative interactions among effectors from *Salmonella* pathogenicity Island-1 (SPI-1, SipA) and *Salmonella* pathogenicity Island-2 (SPI-2 SifA and PipB2), impacting intracellular replication and SCV positioning, providing insights into the spatial dynamics of intracellular replication of *Salmonella* (Brawn et al., 2007).

**FIGURE 2:**
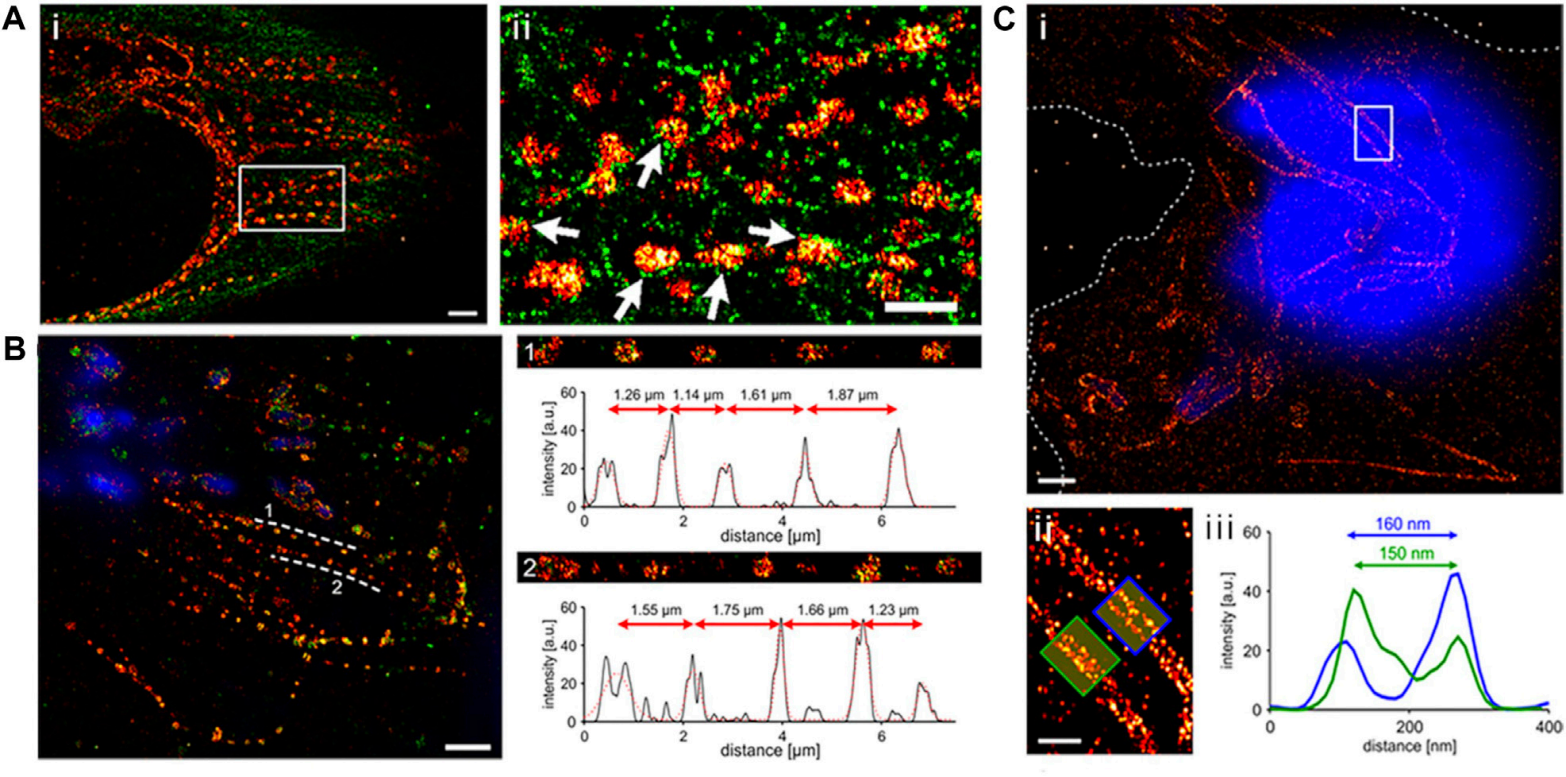
**(A)** Depiction of a dual-color dSTORM image captured from an infected HeLa cell (8 h post-infection). The cell was fixed with PFA and stained to highlight SseJ (red) and tubulin (green). Upon closer examination of the enclosed area in panel A, it becomes evident that SseJ clusters primarily appear in small groupings adjacent to tubulin filaments (indicated by white arrows). Scale bars: 2 μm (panel a) and 1 μm (panels i and ii). **(B)** SseJ clusters exhibit consistent size and regularly spaced arrangements. A typical dSTORM image of a HeLa cell infected with *Salmonella*. Following infection, HeLa cells were fixed using PFA and labeled for SseJ-HA (red) and LAMP-1 (green), with *Salmonella* nucleoids labeled with DAPI (blue). SseJ clusters are arranged in pearls on a string-like fashion (dashed lines indicate this pattern) with a characteristic spacing of 1–2 μm. The straightened trajectory, intensity profiles, and distances between clusters along the dashed lines are displayed on the right (panels 1 and 2). “a.u.” stands for arbitrary units. **(C)** In GA-fixed cells, SseJ appears as continuous filaments. (i) An example image of a HeLa cell infected with *Salmonella* (12 h post-infection), fixed with GA, and stained for SseJ-HA (red) in addition to host and *Salmonella* DNA (blue). Dashed lines delineate the cell boundaries. (ii) A closer view of the enclosed area in panel i showcases *Salmonella*-induced filaments (SIFs). (iii) The cross-sectional intensity profile of the two filaments depicted in panel f reveals SIF diameters of 150–160 nm. Adapted with permission from Gao et al. (2018).

The primary drawback when labeling epitope-tagged effector proteins is the need for fixation and permeabilization in sample preparation before detecting the effectors. This limitation hinders the examination of dynamic processes, offering only a static view of the target molecule at the moment of fixation. It is crucial to note that the fixation procedure itself can potentially disrupt the observed cell structure or the characteristics of the molecule being studied. Such treatments have the potential to alter the shape of cells and create discrepancies in the apparent localization of proteins, resulting in the introduction of artifacts (Ohlson et al., 2008; Schnell et al., 2012; Gao et al., 2018; Singh and Kenney, 2021). Using HA-tagged SseJ produced by *Salmonella*, Gao et al., 2018 investigated the location of this protein within host cells through SMLM. Depending on the fixation technique, SseJ displayed various structural patterns (Figure 2). Within host cells, SseJ formed consistent sized clusters at regular intervals in high osmolality fixative (4% paraformaldehyde, PFA); in low osmolality fixative (0.2% glutaraldehyde, GA), SseJ produced continuous filamentous structures (Figure 2). The clustering of SseJ occurs as a result of the pearling transition. The pearling transition, a form of instability triggered by membranes under tension, is induced by either hypotonic or hypertonic buffer exchange. This process results in the formation of beadlike structures of uniform size and regular spacing (Dabora and Sheetz, 1988). It is noteworthy that high osmolality can also drive the pearling transition. Examples include reshaping of axons during osmotic shock (Pullarkat et al., 2006). The clustering analysis of SseJ indicated the presence of a pearling effect (Figure 2) (Gao et al., 2018). This pearling effect was confirmed by reducing the osmolality of the fixation conditions using glutaraldehyde, which facilitated the visualization of continuous and intact tubules (Figure 2B). The clustered SseJ structures observed in high osmolality fixation were not typical of native structures and were considered as “artifacts” These fixation-induced clusters shed light on the molecular interactions that occur within *Salmonella*-induced filaments (SIFs). For the *Salmonella*-induced filaments to undergo this pearling action, stress had to be applied with force. This force was supplied by kinesin, as demonstrated by two-color super-resolution imaging and subsequent localization analysis, which showed that kinesin co-localized with SifA and SseJ along the SIFs. Consequently, the formation of SIFs was determined to be a force-driven and osmotically sensitive process. Gao et al. (2018).

Epitope tags can be inserted at various positions within a protein open reading frame, including the N- or C-terminus. Selecting the right location is a challenge, as the tag should be accessible for antibody binding without interfering with protein folding or important protein-protein interactions. While it has been demonstrated that the SPI-2 effectors such as SseJ can accommodate an HA-tag at its C-terminus (Chakraborty et al., 2015; Gao et al., 2018), attempts to label additional effectors such as SifA or the substrate-specificity switch SsaP at either their N- or C-termini, or within intracellular loops, while maintaining their functionality, were not successful Brumell et al. (2002b), Gao et al. (2018), Singh et al. (2021). Additionally, it is important to note that epitope tags can undergo post-translational modifications such as phosphorylation, glycosylation, and sulfation in cells. One significant advantage of epitope labelling is its capacity to enhance signal detection from diffusely expressed or low-abundance effectors through secondary or tertiary antibody labeling. For example, signals emanating from chromosomal fusions tagged with epitopes and regulated by endogenous regulatory elements can be readily detected such as PipB2-3XFLAG interactions with kinesin Van Engelenburg and Palmer (2010). Further, the diverse range of epitope tags provides flexibility for multiplex co-localization analysis.

### 3.2 Split‐GFP as an alternative to bulky fluorescent proteins

An alternative method for labeling effector proteins incapable of accommodating large fluorescent proteins involves the utilization of the split-green fluorescent protein (split-GFP) system (Figure 1B). Numerous proteins can be dissected into fragments that possess the inherent ability to spontaneously reassemble, devoid of the need for covalent bonding, resulting in a fully functional protein. In the case of split-GFP, the reassembly of these fragments leads to functional fluorescent proteins, a highly valuable tool for tracking and scrutinizing the localization of translocated effector proteins within host cells. The GFP β-barrel structure is composed of 11 β-strands. In split GFP, these 11 β-strands are segregated into two components: a larger fragment containing the N-terminal 10 β-strands (GFP1-10) and a smaller fragment comprising the C-terminal β-strand (GFP11) Yang et al. (1996), Cabantous et al. (2005), Romei and Boxer (2019). Both of these strands are inherently non-fluorescent in isolation. Prior to infection, the non-fluorescent GFP fragment with strands 1-10 (GFP1–10) is expressed independently within the host cell, while the 11th β-strand of GFP (GFP11) is fused to the effector protein of interest. The chromophore maturation of GFP necessitates the presence of conserved amino acids in strand 11. The recombination of these two fragments spontaneously results in the formation of fluorescent GFP, a process that occurs naturally both *in vivo* and *in vitro*, without the need for additional factors. This method stands as one of the currently available live cell approaches for visualizing translocated effectors within Hela cells during infection, particularly using *Salmonella* effector proteins Van Engelenburg and Palmer (2010), Young et al. (2017).

The Split-GFP system facilitated the observation of *Salmonella* secreted effector proteins, namely, SteA, SteC, and PipB2, in epithelial cells and the macrophage cell line RAW264.7. These results vividly demonstrated the practicality of employing split-GFP to tag various T3SS effectors and monitor the dynamics of effectors within live host cells Van Engelenburg and Palmer (2010), Young et al. (2017). These studies showcased that PipB2 is positioned on highly dynamic tubules (the average speed of SIF growth was ∼0.4 μm/s) that extend from the SCV in both HeLa and macrophage cells, while SteA showed a preference for being concentrated on tubules that co-localized with Golgi markers Van Engelenburg and Palmer (2010). Both effectors played a crucial role in tightly regulating the positioning and maturation of the SCV Van Engelenburg and Palmer (2010), Young et al. (2017). When PipB2-GFP was expressed in host cells ectopically, it accumulated at the perinuclear region and cell periphery. In contrast, PipB2-GFP11, which was translocated by the T3SS, localized within the tubular network Van Engelenburg and Palmer (2010). However, translocation of PipB2-GFP11 was observed only at 4 hours post-infection, whereas immunoblotting of native PipB2 indicated evidence of translocation as early as 2 hours post-infection Szeto et al. (2009), Van Engelenburg and Palmer (2010). This difference can be attributed to the relatively slow maturation process of split-GFP assembly, where it takes an average of 15–30 min for fluorescence complementation to occur. As a result, split-GFP is not the most suitable method for accurately monitoring the real-time movement of effector molecules into host cells. The autofluorescence signal observed during live cell imaging has the potential to disrupt the specific signal emitted by fluorescent labels, reducing the signal-to-background ratio. Furthermore, its applicability is confined to neutral cell compartments, because the fluorescence substantially decreases below pH 6 Campbell (2001). Fluorescent proteins such as mTurquoise2, tagRFP, and mCherry, are characterized by lower pKa values and demonstrate increased resilience against pH variations Thorn (2017). This characteristic makes them a potentially promising substitute for Split-GFP.

The fluorescence signal produced by split-GFP labeled effectors expressed at low levels is quite weak. To overcome this limitation, *Salmonella* SPI-2 effector proteins SseF, SseG, and SlrP, were tagged with 3 × GFP11 to enhance the fluorescence intensity. Nonetheless, given their limited expression levels under native promoters, it was more favorable to adopt an experimental approach involving plasmid-based expression controlled by the more robust *steA* promoter rather than relying on chromosomal tagging. Leveraging signal amplification played a crucial role in facilitating the real-time mapping of effectors localized within infected HeLa cells as well as primary macrophages Young et al. (2017). Recently, the split-GFP system was employed to identify effector proteins AvrB-GFP11 and AvrRps4-GFP11, which are secreted by *Pseudomonas* syringae into subcellular compartments of *Arabidopsis thaliana* plants expressing the GFP1-10 fragment Park et al. (2017). Newly engineered versions of YFP1-10 and CFP1-10 fragments capable of complementing GFP11 are available, giving rise to yellow and cyan fluorescence. Additionally, sfCherry, a derivative of mCherry, can be divided into sfCherry1-10 and sfCherry11 fragments using a method similar to that employed with GFP Kamiyama et al. (2016).

### 3.3 Direct labeling of effectors using a self-labeling tag: the 4Cys‐FlAsH system

To address the challenges arising from bulky fluorescent protein fusions, alternative protein labeling methods utilizing smaller genetically-encoded tags have been devised. One involves the use of a self-labeling protein tag known as the tetracysteine/biarsenical (4Cys-FlAsH) system (Figure 1C) Hoffmann et al. (2010). This approach relies on the interaction between a fluorescein derivative known as fluorescein arsenical hairpin binder (FlAsH) and a short peptide sequence, typically consisting of 12–18 residues, which contain a 4Cys hairpin that is fused to the target effector protein. The core structure of the tetra-cysteine motif is Cys-Cys-Xaa-Xaa-Cys-Cys (CCXXCC), where “X” represents any amino acid, although a preference is often given to Pro-Gly (Adams et al., 2002; Martin et al., 2005). In this arrangement, the peptide forms a hairpin structure that positions the cysteine residues in a manner conducive to interacting with the arsenic atoms present in the fluorescent probe. Subsequently, the effector can be detected by applying the FlAsH dye, which exhibits fluorescence only when it interacts with the 4Cys peptide tag Hoffmann et al. (2010). The FlAsH dye is both fluorogenic and capable of penetrating membranes. The 4Cys-FlAsH system offers an advantage in that it minimally disrupts the structure and function of the target proteins, due to the relatively small size of the peptide tag. This approach has been employed to investigate the real-time localization and secretion kinetics of *Shigella* effectors IpaC and IpaB, as well as *Salmonella* Typhimurium effectors SopE2 and SptP. IpaC and IpaB were distributed diffusely throughout the bacterial cytosol before secretion and swift translocation upon contact with the host cell was observed (Enninga et al., 2005; Van Engelenburg and Palmer, 2008). Approximately 50% of the effectors were translocated within 4 min, where they were localized to actin-rich membrane ruffles adjacent to the invading bacteria Van Engelenburg and Palmer (2008).

Another notable benefit of this approach lies in the utilization of 3 × 4Cys tags, rather than just 4Cys tags, which enhances the binding affinity with the FlAsH dye, resulting in a stronger detected signal. This enhancement effectively overcomes the challenge of low signal intensity, enabling the observation of endogenous chromosomal expression and real-time detection within host cells (Van Engelenburg and Palmer, 2008). While the potential to interfere with the structure and function of the target effector protein is possible, no such effects were observed in the case of *Salmonella* SopE2 or SptP Van Engelenburg and Palmer (2008). SopE2 and SptP exhibited distinctive translocation patterns corresponding to their roles in activating and suppressing the GTPase Cdc42 (Van Engelenburg and Palmer, 2008). The 4Cys-FlAsH technique was significant in probing the rapid kinetics of effector translocation within live host cells.

A significant problem associated with this labeling system is the non-specific labeling of biomolecules rich in thiols. Another significant drawback of this approach is the potential toxicity of biarsenical dyes to eukaryotic cells. While FlAsH concentrations up to 20 μM have minimal impact on bacterial growth and viability, higher concentrations of the dye lead to a dose-dependent reduction in bacterial internalization (Enninga et al., 2005). This labeling system is better suited for studying rapidly translocated effectors (within an hour post-infection). Precise calibration of dye concentration is essential to avoid interfering with the functionality of each effector fusion. The introduction of a red fluorescent form of the FlAsH dye, referred to as resorufin arsenical hairpin binder (ReAsH), enhances the versatility of the labeling process and widens the scope of imaging capabilities to include the orange and red segments of the spectrum. A novel alternative that has been recently developed entails the use of a reagent derived from Rhodamine, known as Rhodamine-derived bis-boronic acid reagent (RhoBo). This reagent selectively binds to tetra-serine motifs and presents advantages such as reduced toxicity and decreased background signals (Halo et al., 2009).

### 3.4 Labeling of effectors using self-labeling enzymes

In this approach, the effector protein of interest is genetically fused with a customized enzyme derivative designed to interact with a synthetic substrate linked to a fluorescent dye (as shown in Figure 1D). One widely used tag in this category is the SNAP-tag (∼20 kDa) (Keppler et al., 2003). It is essentially a modified version of the DNA repair enzyme *O*_6_-alkylguanine-DNA alkyltransferase. The SNAP-tagged protein undergoes covalent labeling with a synthetic fluorescent dye through a fast and precise reaction with small molecules that feature a benzylguanine moiety linked to the fluorescent dye (Figure 1D). A newer variant of this enzyme, the CLIP-tag, is designed to react specifically with *O*_6_-benzylcytosine substrates (Gautier et al., 2008). The HaloTag, derived from a bacterial enzyme haloalkane dehalogenase facilitates the removal of halides from alkyl halides (linked to a fluorescent dye) through nucleophilic displacement (Los et al., 2008). Self-labeling enzymes offer greater specificity when it comes to labeling, but they do come with the drawback of being larger in size. Labeling of SNAP-tags is irreversible and quantitative, making it particularly suitable for identifying and quantifying labeled proteins through in-gel fluorescence scanning of SDS-PAGE gels (Cole, 2013). The use of cell-permeable substrates allows these markers to operate efficiently within cells and within specific subcellular compartments.

Genetic fusions with the SNAP-, CLIP- and Halo-tags coupled with suitable ligands linked to tetramethylrhodamine (TMR), have been invaluable for studying the real-time spatiotemporal localization and translocation of bacterial effector proteins into host cells using super-resolution microscopy (Göser et al., 2019). SPI-1 effectors (SipA, SopB, and SopE) labeled with the HaloTag demonstrated decreased translocation efficiency, whereas fusions with SNAP or CLIP tags exhibited effective translocation. In the case of SPI-2 effectors such as SseJ, SseF, SifA and PipB2, both Halo- and SNAP-tagged fusions showed efficient translocation, while CLIP-effector fusions exhibited lower efficiency (Göser et al., 2019). A limitation is that these tags primarily allow for labeling at the N- or C-termini of proteins, making it challenging to achieve site-specific tagging within internal regions of the protein of interest. Moreover, the relatively significant sizes of the protein tags (Halo-tag, 33 kDa; CLIP-tag and SNAP-tag, ∼20 kDa) can potentially impact the structure and function of the effector protein of interest. Washing steps are often necessary to remove unbound fluorophores to prevent background signals, which can complicate experiments. New fluorogenic labels have been designed that become fluorescent upon labeling, eliminating washing steps. SNAP and CLIP-tags can both be expressed in the same cell and attached to different proteins, facilitating dual-color imaging.

### 3.5 Genetic code expansion as an alternative to classical protein labeling methods

Fluorescent proteins, epitope tags, split-GFP, peptide tags, and enzymes that were discussed in the previous sections have significantly advanced protein labeling techniques for both live and fixed cells. Nevertheless, these tags typically add extra amino acids to either the N- or C-terminus of the effector protein. In many cases, the size of these additional tags is comparable to or larger than that of the effector protein itself. Regardless of their size, these extra tags still have the potential to disrupt the structure and function of the effector and to interfere with the trafficking or post-translational modifications of effector fusion proteins (Akeda and Galán, 2005; Singh et al., 2021; Singh and Kenney, 2021; Singh and Kenney, 2023). To address some of these concerns, we recently employed a new approach. By introducing individual noncanonical amino acids (ncAA) through genetic code expansion (GCE) into target effector proteins, it was possible to incorporate a desired clickable bio-orthogonal functional group precisely at a specific location within the target effector protein (Singh et al., 2021; Singh and Kenney, 2021; Singh and Kenney, 2023). This advancement enabled the visualization of *Salmonella* secreted effectors within host cells while minimizing the potential negative impact of the fluorescent tags, and offered a precise, site-specific labeling method for bacterial effectors.

**FIGURE 3:**
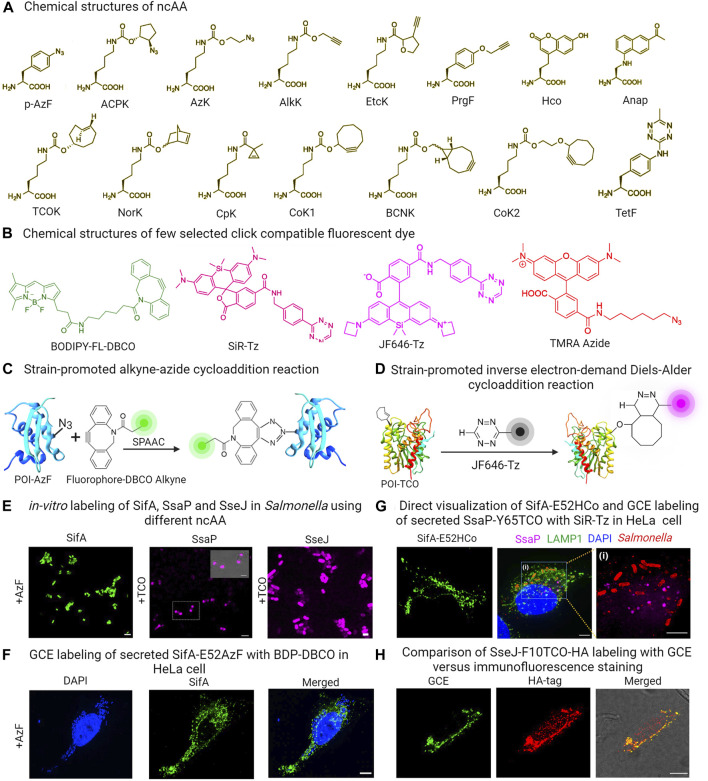
**(A)** Chemical structures of some commonly used ncAAs in GCE labeling **(B)** Structures of GCE click reaction-compatible dyes. **(C)** Schematic representation of effector labeling using a genetically encoded azide-containing protein with a dye containing dibenzocyclooctyne (DBCO) through SPAAC. **(D)** Diagram illustrating the copper-free click reaction of a fluorogenic tetrazine dye, with TCO site-specifically incorporated into an effector via SPIEDAC click reaction. **(E)** *in-vitro* expression of ncAA incorporated SifA-E52AzF, SsaP-Y52TCO and SseJ-F10TCO and subsequent labeling with click reaction enables imaging of labeled proteins in *Salmonella*. **(F)** HeLa cells infected with a *sifA* null strain expressing SifA-E52AzF in the presence of 1 mM AzF enabled visualization of secreted SifA-E52AzF through SPAAC click reaction with BDP-DBCO. **(G)** HeLa cells infected with a *sifA* null strain expressing SifA-E52Hco in the presence of 1 mM Hco, enabled direct visualization of secreted effector without click reactions. HeLa cells infected with an *ssaP* null strain expressing SsaP-Y65TCO was labeled with SiR-Tz. Magnified view of inset indicates secretion of SsaP at later times of *Salmonella* infection. **(H)** HeLa cells infected with *Salmonella* that expressed SseJ-F10TCO-HA in the presence of TCO were tagged with BDP-Tz under physiological conditions. After a quick wash, these cells underwent anti-HA immunofluorescence staining, and images were captured using confocal microscopy. Adapted with permission from (Singh et al., 2021; Singh and Kenney, 2023).

This approach employed a custom-designed orthogonal aminoacyl-tRNA synthetase/tRNA pair that specifically recognized a particular ncAA. Bacteria were then cultured in a medium containing the specific ncAA. When the ncAA enters the bacterial cells and reaches the cytosol, it is recognized and picked up by the orthogonal aminoacyl-tRNA synthetase. This synthetase then attaches the ncAA to the orthogonal tRNA. During the protein translation process, the ribosome utilizes this particular ncAA attached to the orthogonal tRNA to decode a specific designated codon (usually an amber codon, TAG) introduced into the target gene (Chin et al., 2002; Davis and Chin, 2012; Lang and Chin, 2014a; Singh et al., 2021; Singh and Kenney, 2021; Singh and Kenney, 2023). Typically, the amber codon is utilized in this technique, because it is the least commonly used stop codon in bacterial cells. As a result, the ncAA is ultimately incorporated into the growing polypeptide chain as shown in Figure 1E. However, the efficiency of ncAA incorporation depends greatly on the codon selected and its surrounding amino acid sequence. Studies have shown that the most efficient incorporation of ncAA occurs when the preferred codon context is AATTAGACT (Nakamura et al., 2000; Xu et al., 2016). ncAAs are analogs of standard amino acids equipped with a bioorthogonal handle for click chemistry, which enable the GCE technique to introduce a wide range of clickable bioorthogonal handles into the target effector protein (Chin et al., 2002; Davis and Chin, 2012; Lang and Chin, 2014a; Singh et al., 2021; Singh and Kenney, 2021; Singh and Kenney, 2023). These handles can undergo selective chemical labeling with appropriately tailored organic dyes. Figures 3A,B shows some commonly utilized noncanonical amino acids (ncAA) and corresponding organic dyes along with the click reactions used to label bacterial effector proteins. Effector proteins containing clickable bio-orthogonal handles, such as azide, can react with organic dyes containing a dibenzocyclooctyne (DBCO) moiety through a catalyst-free, strain-promoted azide-alkyne cycloaddition (SPAAC; Figure 3C) (Plass et al., 2012; Lang and Chin, 2014a; Lang and Chin, 2014b). Conversely, proteins with bio-orthogonal handles such as ring-strained alkenes, can rapidly interact with tetrazine (Tz)-linked fluorophores through the strain-promoted inverse-electron-demand Diels–Alder cycloaddition (SPIEDAC) reaction, as shown in Figure 3D (Agard et al., 2004; Plass et al., 2012; Lang and Chin, 2014a; Lang and Chin, 2014b). These cycloaddition reactions are suitable for use in live cells and can be rendered fluorogenic. GCE-based protein labeling techniques are not restricted to the N- or C-terminus of the protein of interest. The size of the fluorophore or dye used in this approach is significantly smaller compared to fluorescent proteins, effectively minimizing any adverse impacts on the structure and function of the target effector protein.

Since its inception, numerous sets of orthogonal aminoacyl-tRNA synthetase/orthogonal tRNA pairs have been generated and documented. These pairs have been utilized to label a variety of bacterial proteins, including the cytoplasmic protein Z domain, membrane protein OmpC and intracellular protein LacI in *E. coli* (Zhang et al., 2003; Link et al., 2006; Kipper et al., 2017). HdeA, an *E. coli* acid chaperone, exhibits varying conformations with changing pH levels. To investigate this, Chen and colleagues introduced azido-lysine at a specific site in HdeA, and used pH-sensitive dyes to measure pH differences in distinct local environments and the pH gradient across the cell membrane by placing HdeA in both the cytoplasm and the periplasm (Yang et al., 2014a; Yang et al., 2014b). Zhang and colleagues utilized GCE to illustrate how the targeted acylation of HilA, a bacterial virulence regulator, can reduce infection (Zhang et al., 2020). In a more recent development, we successfully showcased the utility of genetic code expansion in labeling and visualizing the *Salmonella* Typhimurium SPI-2 effectors SifA and SseJ, along with SsaP, the substrate-specificity switch of the T3SS (Singh et al., 2021; Singh and Kenney, 2021; Singh and Kenney, 2023). These proteins had previously proven challenging to visualize using traditional protein labeling methods. SifA is a prominent effector protein central to the development of *Salmonella*-induced filaments (SIFs), a process that promotes intracellular replication and survival of bacterial pathogens. Efforts made in the past to label SifA at either its N- or C-terminus were unsuccessful, as it hindered effector translocation and function. Similarly, labeling SsaP at the C-terminus with PAmCherry, a protein involved in SPI-2 effector secretion regulation, was unsuccessful due to fusion protein cleavage, preventing SsaP imaging (Singh et al., 2021). Nevertheless, we successfully labeled effectors (SsaP, SseJ and SifA) in *Salmonella* and tracked SsaP, SseJ and SifA translocation and localization within infected HeLa cells using GCE (Figures 3E–G) (Ohlson et al., 2008; Singh et al., 2021; Singh and Kenney, 2023). The versatility of GCE as a technique for labeling effector proteins was showcased through two distinct click reactions. In particular, labeling of SifA was accomplished through the utilization of a ncAA-bearing azide functionality, namely, azidophenylalanine (AzF) in conjunction with a dibenzocyclooctyne (DBCO)-containing dye, utilizing the SPAAC click reaction (Figure 3F). In contrast, SsaP was labeled using a trans-cyclooctene (TCO) lysine in combination with tetrazine (Tz)-coupled dyes, employing the SPIEDAC click reaction (Figure 3G) (Singh et al., 2021; Singh and Kenney, 2023). Importantly, in this case, a fluorescent signal was only generated upon successful labeling. The accuracy of SifA, SseJ and SsaP labeling using GCE was verified, and it was ensured that the labeling did not disrupt the function or secretion of these effectors. SifA within *Salmonella*-induced filaments (SIFs) was observed to co-localize with LAMP-1, a well-established marker for the SIFs. Furthermore, SifA interacted with SseJ, a SPI-2 effector, as well as with the motor protein kinesin-1, which plays a role in SIF formation. This discovery shed light on the mechanism behind SIF formation, a force-dependent process (Singh et al., 2021; Singh and Kenney, 2023).

Importantly, labeling specificity was directly compared using the secreted SseJ effector. It was labeled in two ways: the first involved fusion of a C-terminal HA epitope, while the second utilized GCE-labeling using TCO and a water-soluble fluorogenic tetrazine (Tz)-coupled siliconrhodamine (SiR) dye. Although both versions of SseJ showed co-localization, the GCE image exhibited significantly reduced background (see Figure 3D), providing evidence of the superior labeling achieved through GCE, in contrast to immunostaining of small molecule HA tags (Figure 3H) (Singh et al., 2021; Singh and Kenney, 2023). Moreover, labeling SseJ with Janelia Fluor 646 tetrazine (JF646-Tz), a fluorogenic dye compatible with dSTORM, was done without compromising its biological function (Singh and Kenney, 2023). This enabled the use of super-resolution imaging to study the subcellular distribution of *Salmonella* secreted effector proteins in HeLa cells. This approach offers a versatile method for selectively labeling, visualizing, and locating secreted proteins within the host.

Nonetheless, a shared obstacle of several chemical labeling techniques, lies in efficiently introducing appropriately tailored dyes into host cells. This is due to the inherent cellular properties of organic dyes that can hinder the accuracy and effectiveness of labeling. To find an extensive inventory of GCE-compatible dyes that are impermeable or permeable to the cell membrane, please refer to the cited references (Beliu et al., 2019; Meineke et al., 2020; Bessa-Neto et al., 2021; Arsić et al., 2022). To address this issue, we were able to directly visualize the secreted effector SifA by accurately inserting a compact fluorescent ncAA (7-(Hydroxy-coumarin-4-yl) ethylglycine) into the target effector protein (Figure 3G). While fluorescent ncAAs provide an alternative to click reactions, they can only be observed via two-photon microscopy due to their unique excitable nature with UV light. Another limitation of this approach is its low efficiency in incorporating ncAAs. Additionally, it is also imperative to verify the surface accessibility of the chosen ncAA incorporation site for the click reaction by examining the protein structure where possible, as in the case of SifA (Singh et al., 2021). Nonetheless, the approach is applicable to a wide range of T3SS effector proteins. It can also be used to label proteins that are not secreted but pose difficulties for standard labeling approaches.

## 4 Concluding remarks

A hallmark of host-pathogen interactions is the translocation of bacterial effectors into host cells. A range of strategies has been devised to observe and track the secretion and translocation of these effectors. In this review, we summarized principles and applications of some widely used fluorescent labeling methods that facilitate investigating the role of the bacterial effector proteins secreted into host cells. These methods offer valuable insights into the intricate and ever-changing interplay between *Salmonella* (or other pathogens) and its host. However, it is important to grasp the strengths and limitations of each of these methods and employ complementary approaches to ensure a comprehensive understanding of the timing, dynamics, intracellular localization, and potential targets of effector proteins (see Table 1). Despite the rapid advancement in protein labeling techniques, numerous challenges persist in our pursuit of a more accurate and less intrusive illumination of bacterial pathogenesis. Some of the tagging procedures and labeling reactions are incompatible in live systems, limiting their utility for labeling and observing the effector proteins of interest within their native cellular surroundings. Most studies have focused on prominent effectors (such as SipA, PipB2, SopE2 SipA, SopB, SopE, SteA, etc.) with high expression levels, localized within specific sites in host cells. A significant challenge lies in enhancing the sensitivity of these methods to enable visualization of less abundantly expressed effector proteins, while addressing concerns such as photobleaching. Although tandem repeat tags could slightly enhance the sensitivity of some non-abundant effectors, the use of tandem repeat tags carries the risk of increased aggregate formation and potential interference with functionality (Van Engelenburg and Palmer, 2008; Van Engelenburg and Palmer, 2010; Young et al., 2017). These labeling methods also have varying suitability while monitoring effector proteins. For instance, split-GFP is better suited for analyzing effectors that are translocated at later stages, whereas 4Cys-FlAsH is highly effective for tracking rapidly translocated effectors (Enninga et al., 2005; Hoffmann et al., 2010; Van Engelenburg and Palmer, 2010; Young et al., 2017). Most of these methods rely on enhancing signal intensity using plasmid-based expression. This approach is susceptible to introducing experimental artifacts because of effector over-expression and the use of antibiotic selection.

Incorporating ncAA via GCE methods represents the least intrusive approach for obtaining recombinantly labeled proteins. However, there are also notable weaknesses in GCE labeling, particularly when incorporating ncAAs into target proteins. Residual amber-suppressor tRNAs containing a ncAA can lead to off-target protein labeling. Although optimization can mitigate the effects of these background sources, our continued endeavors strive for their eradication. The problem of non-specific incorporation of ncAAs and the low overall labeling efficiencies have both been reported. While the techniques mentioned in this discussion have been significant in examining host-pathogen interactions, there is room for improvement. In summary, it is paramount to conduct a thorough assessment of the appropriateness of the effector labeling technique to attain the best possible outcome, considering the extensive array of available technologies. Although there is no single universally ideal labeling method for all experimental contexts, the selection of a particular labeling approach should be thoroughly examined on an individual basis.
